# Decreased Neuronal Bursting and Phase Synchrony in the Hippocampus of Streptozotocin Diabetic Rats

**DOI:** 10.1155/2014/626108

**Published:** 2014-06-29

**Authors:** Zhimei Qiao, Kangning Xie, Kai Liu, Guoliang Li

**Affiliations:** ^1^Key Laboratory of Brain Functional Genomics (MOE & STCSM), Institute of Cognitive Neuroscience, School of Psychology and Cognitive Science, East China Normal University, No. 3663 North Zhongshan Road, Shanghai 200062, China; ^2^Department of Biomedical Engineering, School of Medicine, Tsinghua University, No. 16 Shuangqing Road, Beijing 100084, China; ^3^School of Biomedical Engineering, Fourth Military Medical University, No. 169 Changle West Road, Xi'an 710032, China; ^4^Department of Anesthesiology, Zhongshan Hospital, Fudan University, No. 180 Fenglin Road, Shanghai 200032, China

## Abstract

Diabetic encephalopathy is one of the complications of diabetes. Cognitive dysfunction is the main consequence. Previous findings from neuroanatomical and *in vitro* electrophysiological studies showed that the structure and function of the hippocampus is impaired in diabetes, which may underlie the cognitive dysfunction induced by diabetes. However the study of electrophysiological abnormality of hippocampal neurons in intact networks is sparse. In the current study, we recorded the spontaneous firing of neurons in hippocampal CA1 area in anesthetized streptozotozin (STZ)-diabetic and age-matched control rats. Profound reduction in burst activity was found in diabetic rats. Compared to control rats, the intra-burst inter-spike intervals were prolonged significantly in diabetic rats, while the burst ratio and the mean number of spikes within a burst decreased significantly. Treatment with APP 17-mer peptide retarded the effects of diabetes on these parameters. In addition, the average PLV of diabetic rats was lower than that of control rats. These findings provide *in vivo* electrophysiological evidence for the impairment of hippocampal function in STZ-diabetic rats, and may have some implications in the mechanisms associated with cognitive deficits in diabetes.

## 1. Introduction

Due to ageing, high calorie diet, and physical inactivity, the prevalence of diabetes mellitus (DM) appears to be rapidly increasing. The term DM describes a metabolic disorder of multiple aetiologies characterized by chronic hyperglycaemia with disturbances of carbohydrate, fat, and protein metabolism resulting from defects in insulin secretion, insulin action, or both [1]. It causes a series of complications including vascular disorder, retinopathy, nephropathy, and peripheral neuropathy which may be disabling or even life-threatening. Currently, the idea that diabetes mellitus has negative impacts on the central nervous system has been widely accepted based on a substantial body of studies [2–7]. Moderate cognitive impairment has been observed in both human beings and animal models with type I or type II diabetes mellitus [5, 8–10]. Recently, diabetes mellitus has attracted considerable attention not only because of its negative effect on the brain but also because of its association with other neurodegenerative diseases [11–14]. Evidence showed that the incidence of Alzheimer's disease (AD) was higher in individuals with diabetes than those without [14, 15].

Streptozotocin- (STZ-) induced rat model is a model of type 1 diabetes mellitus which has been used extensively in studies of the pathophysiology of diabetes [6]. STZ rats show end-organ damage affecting eyes, kidneys, blood vessels, and nervous system. Spatial learning impairment related to the damage of central nervous system has been reported in STZ rats [16, 17]. Although the mechanism underlying cognitive impairment in diabetes is still unclear, accumulating evidence shows that functional or anatomical change of hippocampus is one of those possible mechanisms [18]. As we know, the hippocampus is a critical structure involved in learning and memory in the brain [19]. Several lines of research have studied the effects of experimental diabetes on the synaptic plasticity in the hippocampus. Neuroanatomical research showed that the dendritic morphology of hippocampal neurons was altered in STZ-diabetic rats, including the decrease in the dendritic length and the density of dendritic spines of pyramidal cells [10]. Brain glutamate receptor abnormality was also found in hippocampus of STZ rats [20, 21]. Moreover, the cell proliferation decreased dramatically in the dentate gyrus of STZ-induced diabetic rats [22]. It has been demonstrated that the minor alteration in synaptic efficacy happened earlier than the anatomical abnormality in neurodegenerative disorders [23, 24]. Electrophysiological methods can provide the possibility to detect the alteration in synaptic function earlier, and it will be more valuable in the assessment of the efficacy of therapy. Previous* in vitro* electrophysiological studies have shown that the expression of long-term potentiation (LTP) in hippocampal slices was impaired in diabetic rats, whereas long-term depression (LTD) was enhanced [16, 25]. However, little is known about the* in vivo* electrophysiological changes of hippocampal neurons in diabetes mellitus.

Amyloid precursor protein (APP) is a transmembrane protein expressed in many tissues and concentrated in the synapses of neurons, which plays important roles in the regulation of several important cellular functions, especially in the nervous system, where it is involved in synaptogenesis and synaptic plasticity [26]. APP has six isoforms in central nervous system (CNS), of which APP-695 is the most important [27]. Amyloid precursor protein 17-mer peptide (APP 17-mer peptide) is an active fragment (319–335) of APP-695 in the nervous system that mediates various neuronal activities and functions. It has been reported that APP 17-mer peptide is an effective therapy for diabetes-induced impairment of cognition [28, 29]. APP 17-mer peptide improved the spatial learning and memory when tested by Morris water maze and it increased the synaptic density of diabetic rats. The effect of APP 17-mer peptide on diabetic encephalopathy may be exerted by regulating the metabolism of A *β* [30]. In the present study, the efficacy of APP 17-mer peptide was evaluated by observing its effect on the electrophysiological changes in diabetic encephalopathy.

Here we recorded the spontaneous firing of neurons in area CA1 in STZ-induced diabetic rats and age-matched control rats by* in vivo* extracellular recording, aimed to explore the effects of diabetes on the function of hippocampus. In addition, the efficacy of APP 17-mer peptide was evaluated in this paper. The neuronal firing pattern in the hippocampus was changed in STZ-diabetic rats and APP 17-mer peptide partially reversed the effect of diabetes mellitus.

## 2. Experimental Procedures

### 2.1. Animals

Adult male Sprague-Dawley rats (starting weight ~250 g) were used in this experiment. Animals were housed in a temperature- and humidity-controlled environment and given food and water* ad libitum*. Diabetes mellitus was induced by intraperitoneal injection of streptozotocin (60 mg kg^−1^) (Sigma, St. Louis, MO) dissolved in 0.1 mol/L sodium citrate buffer (PH 4.4). Control rats were injected with saline only. The animal was fasted for 12 hours prior to receiving the injection. Nonfasting blood glucose concentrations of STZ-treated rats and age-matched control rats were measured three days after STZ injection. In all STZ-injected rats, animals with blood glucose level in blood samples obtained by tail prick >15 mmol/L were declared diabetic and selected. All procedures described in this study were reviewed and approved by the Local Institutional Animal Care and Use Committee. All efforts were made to minimize animal suffering and to reduce the number of animals used.

### 2.2. Experimental Design

**Figure 1:**
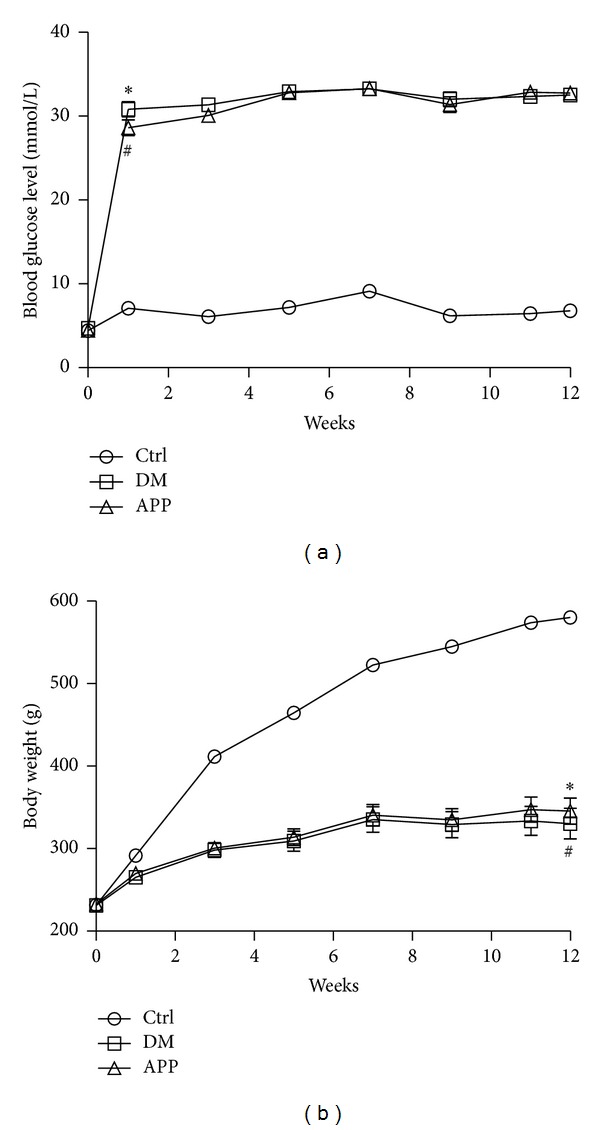
The blood glucose levels and body weights of the rats in Ctrl (*N* = 6), DM (*N* = 6) and APP (*N* = 6) groups. *Significant difference between diabetic rats (both in DM and APP group) and control rats (*P* < 0.05).

Diabetic animals selected into the experiment were divided into 2 groups randomly. One group received subcutaneous injection of APP 17-mer peptide (0.34 *μ*g, daily) in the back of the neck (APP group, *N* = 6). The other group received vehicles alone (DM group, *N* = 6). Another six rats served as age-matched control group (Ctrl group, *N* = 6). The body weights and blood glucose concentrations were measured every two weeks. The changes of body weight and blood glucose level are shown in Figure 1. After 12 weeks, the* in vivo* extracellular recording was performed.

### 2.3. Surgery and Extracellular Recording

The rats were anesthetized with 20% urethane (1.0 g kg^−1^ i.p.) initially. Supplemental doses (1/3 original dose) were given as needed to maintain appropriate level of anesthesia. The depth of anesthesia was monitored by heart rate, respiratory rate, toe pinch reflex, and eye blink reflex. After being anesthetized, the animal was placed in a stereotaxic apparatus. The scalp was removed, and a small bone window (2 × 2 mm) was drilled above the hippocampus (centered at AP: –3.6 mm and ML: +0.22 mm from Bregma, based on the atlas of rat brain [31]) for* in vivo* extracellular recording. Multiunit recording was obtained with 16-channel (2 × 8) polyimide-insulated tungsten microwire arrays (Tucker-Davis Technologies Inc, Alachua, FL, wire diameter 33 *μ*m, electrode spacing 175 *μ*m). After the dura was removed, electrodes then were slowly lowered stereotaxically into CA1 area (~3 mm ventral to dura) by a FHC hydraulic microdrive (FHC, Bowdoinham, ME) until action potentials with pyramidal cell firing characteristics were recorded [32]. A stainless screw placed in the contralateral hemisphere served as ground electrode. The spontaneous firing of neurons (30 min) was bandpass filtered (300–3000 Hz), amplified (RA16PA Medusa PreAmps, TDT), and digitized at 25 kHz with TDT multichannel acquisition system (RX5-2, Tucker-Davis Technologies, Gainesville, FL). For some neurons, wide-band pass (0.5–3000 Hz) signals were recorded and local field potentials were obtained by filtering the raw data with a pass band of 0.5–100 Hz. All the recordings were performed during 1 to 1.5 hours after anaesthesia and in a relatively consistent depth of anaesthesia. The data were then stored in the hard disc for offline sorting and analysis.

### 2.4. Data Analysis

**Figure 2:**
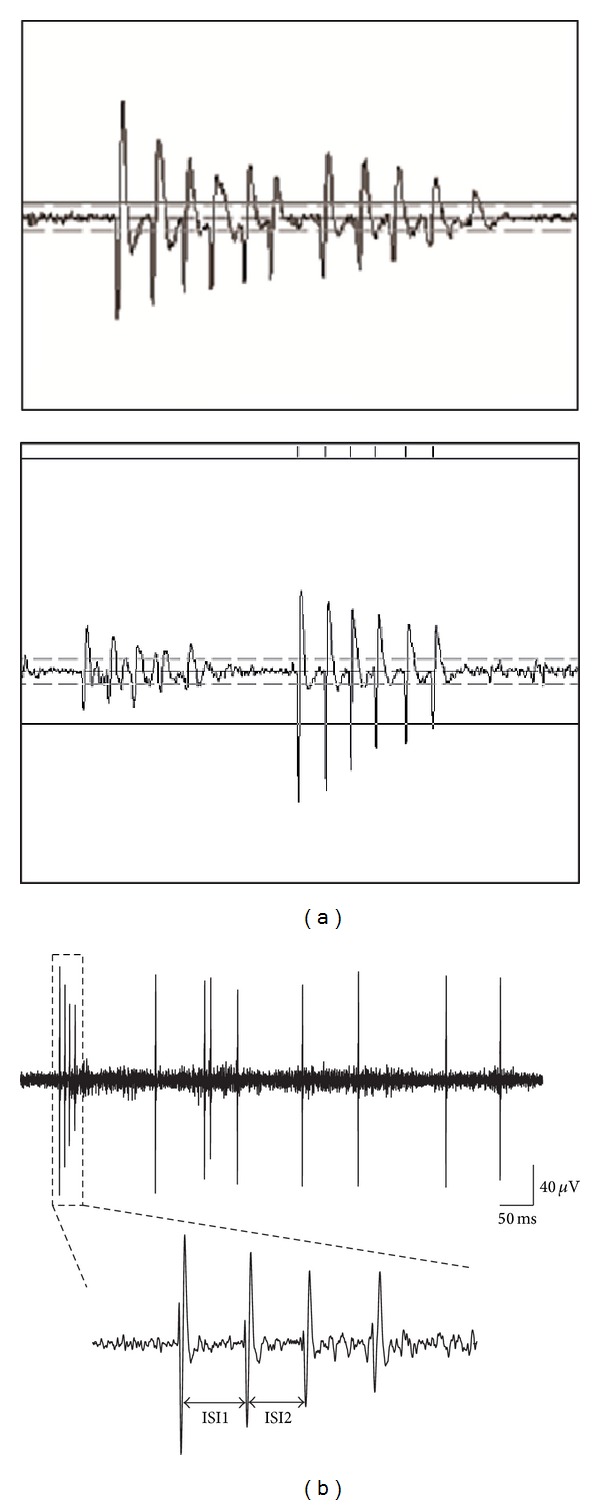
Examples of spontaneous firing of pyramidal neurons recorded from hippocampal CA1 area. (a) Examples of two bursting neurons recorded in one channel. The upper panel showed two bursting neurons whose spike waveforms were too close to be cleanly sorted. The lower panel was an example that the burst can be cleanly sorted to a single unit. (b) An epoch of spontaneous firing which includes a four-spike burst and several single spikes to show the quality of our signal. The burst is exaggerated below. ISI1 is the interval between the second and the first spike while ISI2 is the interval between the third and the second spike.

The spike sorting was performed by principal component analysis in Opensorter software (Tucker-Davis Technologies; Gainesville, FL) and putative single units were isolated by semiautomatic clustering. Neurons with signal-to-noise ratio >3 : 1 were selected to perform further analysis. Autocorrelograms and other sequential analyses were calculated using custom software (developed using MATLAB, Mathworks, MA). Putative pyramidal cells and interneurons were distinguished by the spike duration [33], discharge frequency [34], and autocorrelation function [35]. Those neurons with spike duration >0.5 ms, background firing rate <10 Hz, and a peak at 3–5 ms in autocorrelogram were classified as pyramidal neurons. Putative pyramidal cells were classified into bursting cells and nonbursting cells. Complex spike bursting is characteristic of pyramidal cells in the intact hippocampus. It has been demonstrated that compared to single spikes, bursts have a special role in synaptic plasticity [36]. To explore the changes of synaptic plasticity in diabetes, we mainly analyzed the burst firing pattern in this study, based on the hypothesis that impairment of synaptic plasticity in diabetes should have representation in changes of burst features. A burst is identified by the criteria that it consists of 2–6 successive spikes with short interspike intervals (⩽6 ms) and decreasing amplitude [32]. To reduce the error rate in spike sorting when two or more bursting neurons were recorded from one channel at the same time, we abandoned those channels which had two or more bursting neurons difficult to be cleanly sorted (Figure 2(a), upper panel). Further analysis including burst ratio (defined as bursts per 500 spikes), mean number of spikes in bursts, intraburst interspike intervals, and spike amplitude attenuation within bursts were performed then. For intraburst interspike interval, only those neurons with number of spikes within burst >3 were used for analysis, and only peak-peak intervals between the first and second spikes (ISI1) and the second and the third spikes (ISI2) were calculated. Spike amplitude attenuation within a burst was determined by dividing the amplitude of the second spike (or third spike) in the burst by the amplitude of the first spike. To make sure that a burst is coming from a single neuron, we strictly inspected the burst selected for further analysis. For a putative bursting neuron, first, we visually selected 20 bursts with very similar shapes as standard bursts. Then, we calculated the mean ± SD of ISIs and attenuation of amplitudes of those 20 bursts and automatically selected those bursts whose intraburst ISIs and the attenuation of amplitudes are very close to standard bursts (≤ mean ± 2 SD) by a program for further analysis.

To explore the changes of temporal coordination in diabetic rats, the degree of phase-locking between neuronal spike firing and LFP delta oscillation was calculated. The delta oscillation was obtained by band-pass filtering the hippocampal LFP into band frequency 1–4 Hz, and the filtered delta band was decomposed into phase Φ(*t*) component by using the Hilbert transform [37]. The phase value of a spike was determined by the instantaneous phase value of delta wave at the time when the spike occurred. For further quantifying the degree of spikes' phase locking to hippocampal delta rhythm, we used the phase-locking value (PLV) which is defined as [38]

(1) $$\begin{array}{c} \text{PLV}=||\frac{1}{N}\overset{N}{\underset{i=1}{\sum }}e^{j\phi_{i}}||, \end{array}$$

where *ϕ*_*i*_ is the phase value of the *i*th spike, while *N* is the number of spikes. PLV was compared between control and diabetic rats. Meanwhile, the spike train of individual unit was broken up at the troughs of the hippocampal delta rhythm and the resulting segments were stacked up to form the delta-triggered rasters (Figure 6(b), upper panel). The lower panels of Figure 6(b) were the corresponding phase value distributions.

### 2.5. Statistical Analysis

Data was expressed as means ± standard error of mean (SEM). One-way repeated measures analysis of variance (ANOVA) and Tukey's post hoc test were used to analyze blood glucose level, body weight, and the burst ISI data. Statistical comparison of burst ratio and number of spikes within bursts was made by applying a one-way Kruskal-Wallis test and post hoc analysis with the Mann-Whitney test. Difference in the number of bursting and nonbursting pyramidal cells among the three groups was calculated by chi-square method. If there was statistical significance, further analysis was made to compare the difference between groups by partition of chi-square test. Student's *t*-test was performed to compare the phase-locking values between control and DM groups. *P* < 0.05 was considered statistically significant.

### 2.6. Histology

After completion of the electrophysiological recording, lesion was made at all the tips of all electrodes by passing direct current (10 *μ*A, 10 s). Then the rats were deeply anesthetized and perfused intracardially with 100 mL of 0.9% saline solution followed by 300 mL of 4% buffered formalin phosphate solution. The brains were sectioned coronally at 40 *μ*m thickness. The sections were mounted on slides and stained with hematoxylin and eosin for localizing the electrode tip.

## 3. Results

### 3.1. Blood Glucose Levels and Body Weight

There were no differences in body weight and blood glucose levels before the administration of STZ among the three groups (Figure 1). However, three days after STZ administration, the blood glucose levels of diabetic rats (30.8 ± 1.6 mmol/L for DM group and 28.6 ± 1.6 mmol/L for APP group) were significantly higher than that of the control rats (7.1 ± 0.3 mmol/L, *P* < 0.0001) and maintained a higher level until the start of the electrophysiological recording. The blood glucose levels of control rats fluctuated within a narrow range during the observation period. As expected, body weight of the control rats increased steadily during the observation period while that of the animals in the other two groups increased slowly. Twelve weeks after the STZ administration, body weights of rats in DM (334 ± 31 g) and APP groups (347 ± 26 g) were significantly lower than that of control rats (574 ± 9 g, *P* < 0.0001). There were no significant differences in body weight and blood glucose levels between rats in DM and APP group. The data of blood glucose levels and body weight reported in this paper are consistent with previous reports [10, 39].

### 3.2. Changes of Burst Firing Pattern

Data from electrodes falling outside hippocampal CA1 area were excluded from dataset. A total of 210 neurons were recorded from area CA1. The numbers of different types of neurons in control, DM, and APP group are shown in Table 1. The detected bursting neurons in the control group were significantly more than in the other two groups.

**Figure 3:**
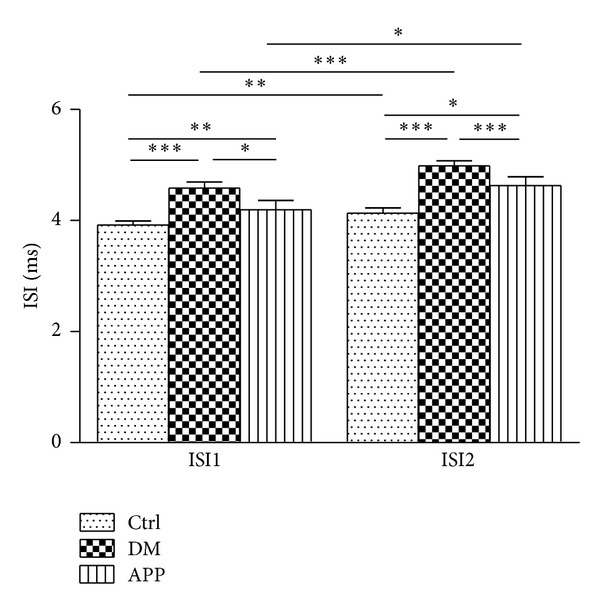
The intra- and intergroup comparison of intraburst ISI1 and ISI2. ISI1 and ISI2 of neurons (*n* = 59) recorded from DM rats were much longer than that of control rats (*n* = 26). APP 17 peptide treatment significantly decreased neuronal intraburst ISI1 and ISI2 (*n* = 35) compared with DM rats, while significant differences were also found between rats in control and APP group. In addition, ISI2 was a little longer than ISI1 in all the three groups (*P < 0.05; **P < 0.01; ***P < 0.001, Mann-Whitney test). “*n*” represents number of neurons.

An epoch of spike trains including isolated spikes and bursts recorded from CA1 area is shown in Figure 2. For bursting neurons, ISI1 and ISI2 were compared among groups. As shown in Figure 3, ISI1 of rats in DM group (4.58 ± 0.1 ms; *n* = 26) was significantly longer than that in control group (3.72 ± 0.05 ms; *n* = 59; *P* < 0.001) and APP group (4.15 ± 0.15 ms; *n* = 35; *P* < 0.01). There was also significant difference between ISI1 of control group and that of APP group (*P* < 0.05). For ISI2, the same difference was also observed between DM group (5.02 ± 0.09 ms; *n* = 26) and control group (3.94 ± 0.07 ms; *n* = 59; *P* < 0.001) as well as DM group and APP group (4.32 ± 0.17 ms; *n* = 35; *P* < 0.001). ISI2 of APP group was slightly longer than that of control group (*P* < 0.05). In addition, the comparison between ISI1 and ISI2 among the three groups showed that ISI2 was significantly longer than ISI1 in all the three groups (Figure 3).

**Figure 4:**
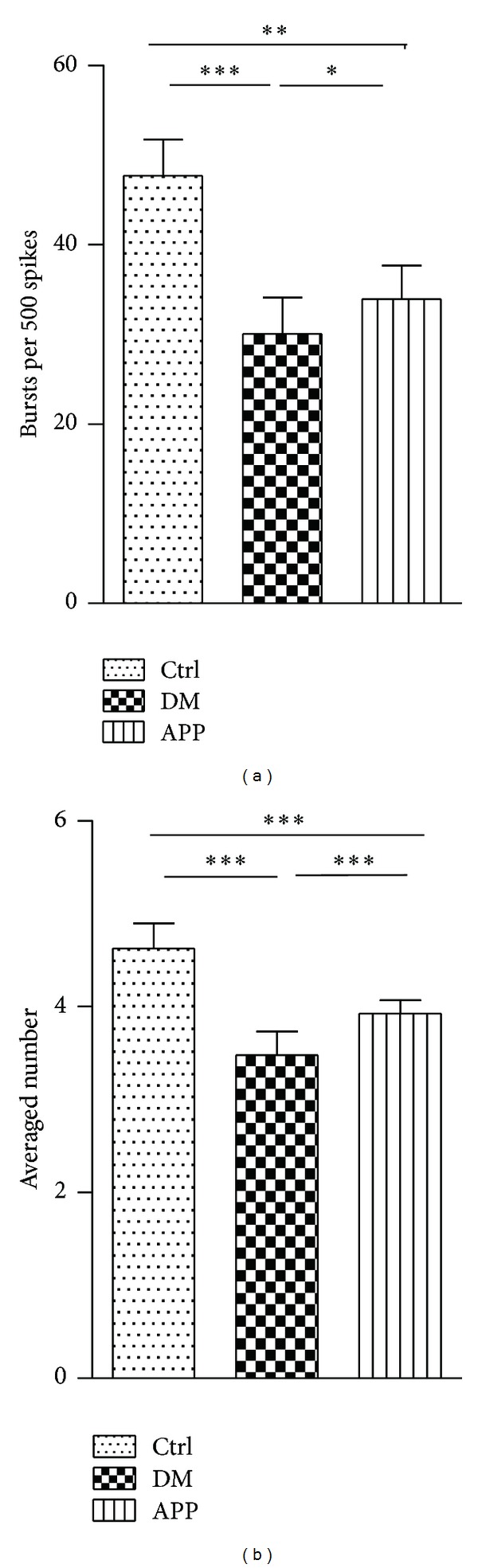
Comparison of burst ratio and number of spikes within burst between groups. The burst ratio of neurons from diabetic rats (*n* = 26) is significantly lower than that of control rats (*n* = 59, *P* < 0.001, Mann-Whitney test). Decrease of the number of spikes within bursts in diabetic rats was also observed (*P* < 0.001, Mann-Whitney test). Compared to that of diabetic rats, APP 17-mer peptide increased the burst ratio and number of spikes within bursts (*n* = 35, *P* < 0.05 for burst ratio; *P* < 0.001 for number of spikes within bursts, Mann-Whitney test) but still had significant difference with control rats (*P* < 0.01, Mann-Whitney test, for burst ratio; *P* < 0.001 for number of spikes within bursts). “*n*” represents number of neurons.

To describe the burst rate during the recording period, we used the burst ratio as one index of burst propensity, which was calculated by the number of bursts per 500 spikes. The mean burst ratio of control group (47.7 ± 4.0; *n* = 59) was significantly higher than that of DM group (30.1 ± 4.1; *n* = 26; *P* < 0.05) and APP group (33.9 ± 3.8; *n* = 35; *P* < 0.05) (Figure 4(a)). A significant difference was also found in burst ratios between DM group and APP group (*P* < 0.05). Our results suggested that APP 17-mer peptide partially reversed the decreased burst ratio in diabetic rats.

In addition, we also analyzed the difference in number of spikes within bursts between groups (Figure 4(b)). In general, the mean of spike counts of control rats (4.6 ± 0.3; *n* = 59) was a little larger than that of DM group (3.5 ± 0.3; *n* = 26; *P* < 0.05). Treatment of APP 17-mer peptide had an effect on the decreased number of spikes within bursts observed in diabetic rats. APP 17-mer peptide increased the reduced spike number to 3.9 ± 0.1 (*n* = 35; *P* < 0.05) that was observed in diabetic rats to some degree but a difference from control rats was still observed (*P* < 0.05).

**Figure 5:**
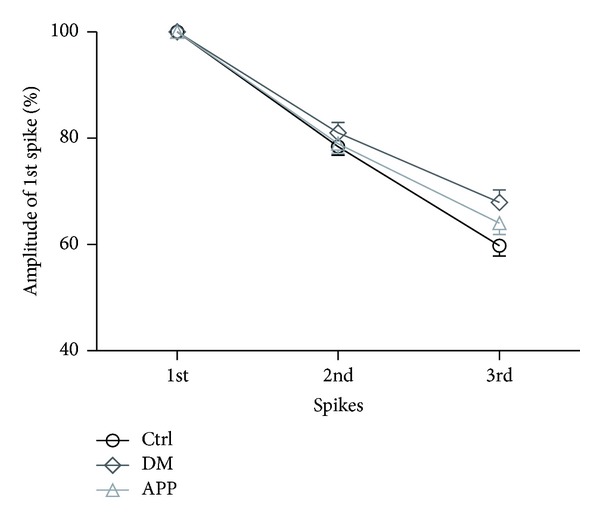
Comparison of intraburst amplitude attenuation between the three groups. Amplitude attenuation within bursts was reduced in diabetic rats (*n* = 26) either for the 2nd/1st spike (*P* < 0.001, Mann-Whitney test) or for the 3rd/1st spike (*P* < 0.001, Mann-Whitney test). Treatment of APP 17-mer peptide partially reversed the decrease in amplitude attenuation (*n* = 35, *P* < 0.001, Mann-Whitney test, for the second spike; *P* < 0.001 for the third spike). The difference in amplitude attenuation between APP and Ctrl group (*n* = 59) only was significant at the third spike (*P* < 0.001, Mann-Whitney test). For the second spike, no difference was observed (*P* = 0.314, Mann-Whitney test). “*n*” represents number of neurons.

Decreasing amplitude is a prominent characteristic of burst firing. Analysis of the spike amplitude attenuation within bursts showed that, for the 2nd spike, there was a decrease in amplitude attenuation in diabetic rats (0.81 ± 0.01) compared with control rats (0.78 ± 0.02, *P* < 0.05, Figure 5). The decrease of amplitude attenuation was more apparent in the third spikes (0.59 ± 0.02 for Ctrl group; 0.68 ± 0.02 for DM group, *P* < 0.05). For the third spike, treatment of APP 17-mer peptide partially reversed the decrease of amplitude attenuation found in diabetic rats (0.64 ± 0.02 for APP group, *P* < 0.05).

### 3.3. Impaired Temporal Coordination Measured by Phase Locking Value

**Figure 6:**
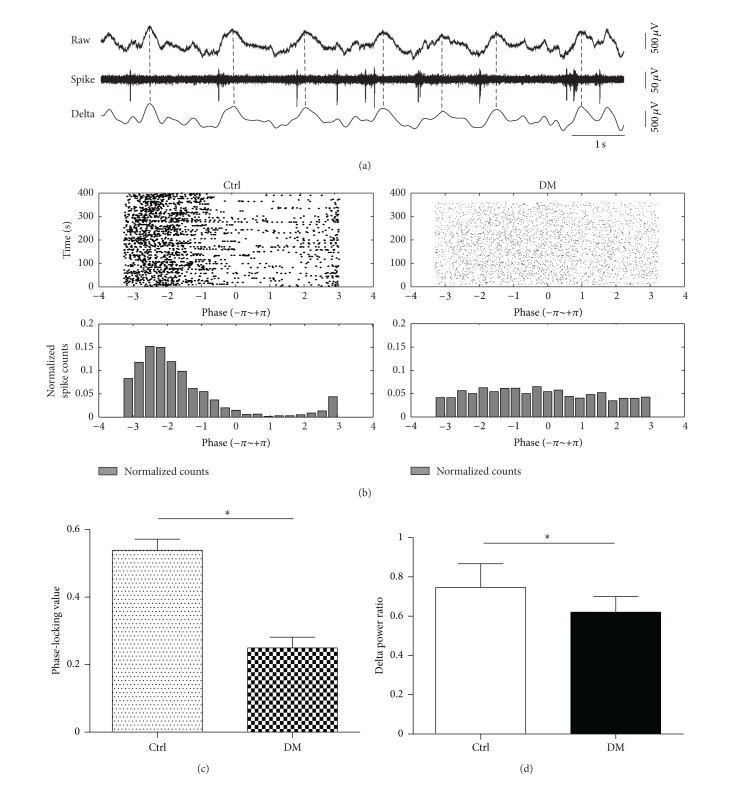
Phase-locking of hippocampal neurons. (a) Epoch of wide-band raw trace as well as corresponding delta oscillation of LFP (band-pass filtered in the frequency band of 1–4 Hz) and spike train. The phase-locking between spike and delta oscillation of LFP is apparent. (b) Phase value rasters (upper panel) and the corresponding phase value (lower panel) distributions of two typical neurons in the control and DM groups. Control rats showed stronger phase-locking phenomenon than diabetic rats. (c) Comparison of phase-locking values between diabetic and control rats. The PLV in control rats (*n* = 59) was significantly higher than that in diabetic rats (*n* = 26) (*P* < 0.05, Student's *t*-test). (d) Comparison of delta power ratio between Ctrl and DM groups. There was significant difference between Ctrl (*N* = 6) and DM (*N* = 6) groups (*P* < 0.05, Student's *t*-test). “*N*” represents number of rats and “*n*” represents number of neurons.

To investigate the change of temporal coordination in diabetic brains, phase-locking analysis between spike and local field potential (LFP) was performed. Delta waves were broadly observed in the recorded LFPs in all rats of DM and control groups. Analysis of the power of LFP showed that low-frequency, high-amplitude waves peaking between 1–4 Hz are the dominant activity pattern, which have been reported often during deep slow wave sleep and anesthesia [40, 41]. A typical example of phase-locking phenomenon was shown in Figure 6(a). The raw trace was filtered into delta oscillations (1–4 Hz) and spikes. Spikes were phase-locked to the trough of the delta waves. Figure 6(b) shows the phase value raster (upper panel) and corresponding phase value distribution (lower panel) of two typical neurons in control and DM rats. The difference in the degree of phase locking between control and DM group is shown in Figure 6(c). There was significant difference between PLV of control rats (0.54 ± 0.03) and that of DM rats (0.25 ± 0.03, *P* < 0.001). Our results showed that the phase-locking between spikes and delta oscillations in DM rats was poorer than that in control rats. To investigate if the network oscillation of DM rats is generally different from that of control rats, we compared the power of LFP activity between control and DM groups. Delta power ratio (delta power ratio was calculated by ratio of power in LFP frequency band 1–4 Hz to that in the whole LFP band (0.5–100 Hz)) was compared between neurons recorded from control and DM groups (Figure 6(d)). Our data showed that, compared to that of Ctrl group (0.75 ± 0.12, *N* = 6), delta power ratio decreased significantly in DM group (0.62 ± 0.08, *N* = 6, *P* < 0.05).

## 4. Discussion

In the present work, we used* in vivo* multichannel electrophysiological method to study the effect of diabetes mellitus on the spontaneous activity of hippocampal neurons in STZ-diabetic rats. Our results demonstrate that burst activities of hippocampal pyramidal neurons were impaired in STZ-diabetic rats, including decreased number of bursting neurons, prolonged intraburst interspike intervals, decreased burst ratio, and the number of spikes within a burst. APP 17-mer peptide can partially reverse the observed negative effect. Meanwhile, the phase synchrony between control and diabetic rats was also analyzed. Phase-locking value between single neuronal spiking and delta oscillation (1–4 Hz) of local field potential was significantly decreased in diabetic rats. The changes observed in this work may be correlated with the impairment of cognitive function in STZ-induced diabetes.

The dramatic reduction in burst activity defines possible mechanisms that may underlie the cognitive impairment observed in diabetes. Specifically, bursts as a unit of neural information have been postulated to play important roles in brain function [36, 42–44]. Compared with single spikes, presynaptic bursting of action potentials can be more reliably transmitted to the postsynaptic neurons and increase the probability of information transmission between neurons [45, 46]. Moreover, several lines of evidence indicated that bursts have a special role in synaptic plasticity [36]. Bursts may enhance synaptic plasticity at both presynaptic inputs that initiate a burst [36, 47] and postsynaptic potentiation caused by supralinear summation of excitatory postsynaptic potentials (EPSPs) at postsynaptic targets [48, 49]. It has been shown that the processes of synaptic modification seemed very sensitive to the types of bursts. The number of spikes within burst was related to the degree of synaptic modification. Bursts with two spikes produce little synaptic modification, and bursts with three spikes induce some LTP or LTD, while bursts with four spikes induce nearly maximal LTP or LTD [44]. Thus, the decreased number of spikes within burst in diabetic rats would be expected to decrease the synaptic efficiency.

A very interesting finding in the present study is that the intraburst interspike intervals in diabetic rats are significantly longer than those in control rats. It has been proposed that the fine temporal structure of burst has role in neural computation when bursts are analyzed as unitary events [50, 51]. Changes in the interspike interval may have pronounced influence on synaptic modification. Whether in the protocol of LTP elicited by high-frequency stimulation or primed burst potentiation elicited by burst stimulation, or short-term facilitation elicited by paired pulse stimulation, the interstimulus intervals have enormous effect on the postsynaptic responsive efficacy [52]. Previous studies observed the effects of complex spike trains in LTP/LTD induction by using paired bursts of spikes with varying pre/postintervals and burst frequency [53, 54]. Their results showed that in addition to pre/postinterval, synaptic modification was also found to depend on the firing frequency within each burst. The magnitude of LTP increases with the burst frequency. It is often assumed that the shorter the interspike interval within the burst, the better: the summed postsynaptic potential activated by presynaptic bursting of spikes is larger when the interval between the spikes is smaller [55].

Exactly how the STZ treatment came to reduce the burst activity is unknown but could involve alteration in presynaptic afferent fiber or changes in transmitter release. The decrease of NMDA receptor may be one of the possible causations. It is generally thought that bursting arises from activation of NMDA receptors and opening of L-type calcium channels. Thus, decrease in NMDA receptors will lead to a lack of sustained NMDA receptor-mediated membrane depolarization and then results in the decreased probability of successive action potential generation and the reduction of the number of closely spaced spikes and burst activity [56–58]. It has been shown that, after 12 weeks of STZ-diabetes, the level of the NR2B subunit of the NMDA receptor is decreased by 40%. Moreover, the phosphorylation of the NR2A/B subunits by Ca2+/calmodulin-dependent protein kinase II is reduced in STZ-diabetic rats [39]. Our results are in line with the* in vitro* electrophysiological findings that the expression of NMDA-dependent LTP in the CA1 is impaired in diabetes [5, 59]. Further investigation will be required to clarify mechanism underlying the reduction of burst activity in diabetes.

Oscillatory activity plays a crucial role in hippocampal functions that are mainly expressed during diverse behaviors and various stages of sleep. Delta oscillation appears as a large-amplitude slow frequency (1–4 Hz) extracellular rhythm of local field potentials during deep slow-wave sleep as well as under urethane anaesthesia, which is believed to be important in enhancing synaptic excitability in the hippocampus [60, 61]. In this paper, delta power ratio was found to be significantly decreased in diabetic rats. It is possible that the differences in bursting behavior observed in this paper may be due to the differences in network states between the two groups. More comprehensive experiment should be designed to testify this hypothesis in future study. Phase-locking analysis showed that PLV of diabetic rats was significantly lower than that of control rats, which demonstrated that STZ-induced diabetes impairs the temporal coordination between neuronal spiking and slow oscillation of population activity in the hippocampus. The possible explanation for the disruption of neural coordination is the loss of neurons [62] and dendritic spines [10] in diabetic brains leading to the alteration in the capacity of individual neuron receiving inputs from cell assemblies. Thus, the coordination between the firing of single neuron and the activity of cell assemblies is disrupted. Recently, Sigurdsson et al. proposed that the impaired hippocampal-prefrontal synchrony may be a fundamental component of the pathophysiology underlying schizophrenia [63]. Our study provided further evidence for the theory that abnormal neural synchrony may be one of the mechanisms underlying cognitive dysfunction related to brain disorders.

Application of polypeptide-based therapy against neurodegenerative diseases has been reported in the past [64–67]. It is a promising field of treatment for neurodegenerative diseases. Previous studies reported that amyloid precursor protein (APP) is a protein with neurotrophic function [28]. APP 319-335 segment (APP 17-mer peptide) of APP 695 was demonstrated to retard neuronal degeneration in diabetic mice by regulating the metabolism of A*β* [30]. At the behavioral level, when spatial learning and memory were tested in water maze, APP 17-infused diabetic rats showed increased memory retention [28]. In the present paper, the degree of firing pattern changes in diabetic rats with APP 17-mer peptide treatment was smaller than that in diabetic rats, which implied that treatment of APP 17-mer peptide can retard the degeneration induced by STZ diabetes.

In summary, our findings describe* in vivo* electrophysiological observations of hippocampal neuronal activity in STZ-diabetic rats. We found that neuronal burst activity was significantly reduced in diabetic rats. From the perspective of local circuit, phase synchrony was also impaired by STZ treatment. These alterations may be responsible for the impairment of hippocampal function in STZ-induced diabetic rats. Our results at least partly imply the mechanisms underlying the cognitive deficits in diabetes.

Our study has limitations in the following two aspects. (1) The electrophysiological recording was performed on anesthetized rats. Because diabetic rats may have altered metabolism and blood brain barrier, the anesthetics may have different effect on control rats and diabetic rats. To minimize the different effect of anesthetics, the dose of anesthetics was reduced slightly (about 1/10) in diabetic rats. At the meantime, we visually inspected respiration rate and heart rate carefully and frequently during the experiment to make sure that the respiration and heart rate were maintained in a consistent level among the three groups. Even so, we cannot completely exclude the possibility that the results reported in this paper were partly due to the effect of anesthesia. However, our results showed that there was no difference in blood glucose levels and body weight between diabetic rats and diabetic rats with APP 17-mer peptide treatment, and the same dosage of urethane was delivered to the two groups, while the changes of firing pattern in the two groups were different significantly. Our results strongly implied that the changes of firing pattern were the consequence of STZ-induced diabetes. The conclusion will be testified in the future work on awake rats. (2) The microelectrode used in this study was multielectrode arrays rather than tetrode. Due to the dense distribution of neurons in hippocampus, it is hard to cleanly sort signals recorded from one single electrode into single units. However, considering that our data has high SNR and we abandoned that the neurons cannot be cleanly sorted, we believe that the error in sorting was controlled in an acceptable range.

## Figures and Tables

**Table 1:** Number of different types of cells recorded from CA1 in the three groups.

Group	Pyramidal cell		Interneurons	Total
	Bursting	Nonbursting		
Ctrl	59	12	6	77
DM	26	38∗	3	67
APP	35	23^#$^	8	66

Chi-square test showed that there was significant difference (*χ* ^2^ = 29.101, *P* < 0.001) among groups. Further partitioning chi-square analysis showed that there was also significant difference between groups (∗DM versus Ctrl, *χ* ^2^ = 26.039, *P* < 0.001; ^#^APP versus Ctrl, *χ* ^2^ = 8.36, *P* < 0.01; ^$^APP versus DM, *χ* ^2^ = 4.733, *P* < 0.05).
